# O_3_-Induced Leaf Senescence in Tomato Plants Is Ethylene Signaling-Dependent and Enhances the Population Abundance of *Bemisia tabaci*

**DOI:** 10.3389/fpls.2018.00764

**Published:** 2018-06-12

**Authors:** Honggang Guo, Yucheng Sun, Hongyu Yan, Chuanyou Li, Feng Ge

**Affiliations:** ^1^State Key Laboratory of Integrated Management of Pest Insects and Rodents, Institute of Zoology, Chinese Academy of Sciences, Beijing, China; ^2^College of Life Sciences, University of Chinese Academy of Sciences, Beijing, China; ^3^State Key Laboratory of Plant Genomics, National Center for Plant Gene Research, Institute of Genetics and Developmental Biology, Chinese Academy of Sciences, Beijing, China

**Keywords:** elevated O_3_, ethylene, *Bemisia tabaci*, leaf senescence, amino acid, hormone-dependent defense

## Abstract

Elevated ozone (O_3_) can alter the phenotypes of host plants particularly in induction of leaf senescence, but few reports examine the involvement of phytohormone in O_3_-induced changes in host phenotypes that influence the foraging quality for insects. Here, we used an ethylene (ET) receptor mutant *Nr* and its wild-type to determine the function of the ET signaling pathway in O_3_-induced leaf senescence, and bottom-up effects on the performance of *Bemisia tabaci* in field open-top chambers (OTCs). Our results showed that elevated O_3_ reduced photosynthetic efficiency and chlorophyll content and induced leaf senescence of plant regardless of plant genotype. Leaf senescence in *Nr* plants was alleviated relative to wild-type under elevated O_3_. Further analyses of foliar quality showed that elevated O_3_ had little effect on phytohormone-mediated defenses, but significantly increased the concentration of amino acids in two plant genotypes. Furthermore, *Nr* plants had lower amino acid content relative to wild-type under elevated O_3_. These results provided an explanation of O_3_-induced increase in abundance of *B. tabaci.* We concluded that O_3_-induced senescence of plant was ET signal-dependent, and positive effects of O_3_-induced leaf senescence on the performance of *B. tabaci* largely resulted from changes of nutritional quality of host plants.

## Introduction

Global tropospheric ozone (O_3_) concentration has increased from pre-industrial less than 10 to current 35–50 ppb in the Northern hemisphere (Ainsworth et al., 2012), and is predicted to be still increasing at a rate of approximately 0.5–2% per year in some regions, such as East Asia (Ohara et al., 2007; IPCC, 2013; Cooper et al., 2014). Tropospheric O_3_ is an important atmospheric pollution type and also a greenhouse gas, which can cause changes in plant metabolism, such as changes in photosynthetic rate, nutritional content, and secondary compounds (Ashmore, 2005; Gupta et al., 2005). The alteration of plant biochemistry under elevated O_3_ could affect the quality and palatability of plant tissue, and therefore changes in interactions with herbivorous insects (Peltonen et al., 2010).

Elevated O_3_ leads to significant changes in plant phenotypes, such as visible leaf injury, acceleration of leaf senescence, and growth limitation (Miller et al., 1999; Wittig et al., 2007), with considerable concern on leaf senescence. Elevated O_3_ causes a series of senescence-related processes which includes decrease in photosynthetic rate, damage in chlorophyll fluorescence, and increase in leaf defoliation (Gielen et al., 2006). Ethylene (ET) signaling pathway is widely accepted as a positive mediator of developmental leaf senescence, in which leaf senescence is delayed or alleviated for ET-insensitive mutants, and accelerated for plants exogenous application of ET (Lim et al., 2007; Koyama, 2014; Qiu et al., 2015). Recent research demonstrated that ET signaling pathway also serves as a positive mediator in abiotic stress-induced leaf senescence, such as drought or heat stress (Young et al., 2004). High temperature-induced leaf senescence is delayed by spraying ET inhibitor 1-MCP in soybean plants (Djanaguiraman et al., 2011). Although ET production and its signaling pathway are upregulated under elevated O_3_ (Moeder et al., 2002; Ludwików et al., 2009, 2014), it is unclear the role of ET signaling pathway in O_3_-induced leaf senescence.

There are inconsistent responses of insects to elevated O_3_ (Manninen et al., 2000; Percy et al., 2002; Cui et al., 2012), of which factors have been the focus of study, particularly, response of host plants, sensitivity and parameters of herbivores, or O_3_ level (Holopainen and Kainulainen, 1997; Hillstrom et al., 2010; Couture and Lindroth, 2012). These reports suggest that the variable responses of N nutrition and secondary metabolites in host plants under elevated O_3_ result in the contradictory effects on herbivorous insects. The population fitness of herbivores tends to be reduced if host plants have low N nutrient value and high level of defense metabolites, while it tends to be increased on host plants with high N nutrition under elevated O_3_ (Holopainen and Kainulainen, 1997; Cui et al., 2012). It is worthwhile to note that Holopainen (2002) has proposed a hypothesis that contradictory impacts could be interpreted by premature senescence of host plants under elevated O_3_, but experimental evidence is lacking.

Leaf senescence indeed affects the performance of insects via an alteration of plant N nutrient value and defense metabolism. With respect to nutritional value, senescing leaves may serve as a good source of nitrogen for sap-sucking insects. The N-containing substances are converted into amino acids and transported from the senescing leaves via phloem loading (Lim et al., 2007). During export from senescing leaves via phloem, nitrogen is easily accessed by sap-sucking insects. For example, the leaf senescence induced by black pecan aphid (*Melanocallis caryaefoliae*) infestation increases amino acid concentrations in phloem (Cottrell et al., 2009, 2010), which may be responsible for promoting subsequent aphid setting and nymphal development (White, 2015). In addition to nutrient metabolism, premature leaf-senescence can positively regulate plant resistance against sap-sucking insect infestation. Green peach aphid (*Myzus persicae*, GPA) counts are reduced on hyper-senescence mutant plants (*cpr5* and *ssi2*), while increase in *pad4* mutants is observed with a delay in GPA-induced senescence (Pegadaraju et al., 2005). Therefore, although needing experimental testing, it is reasonable to speculate that O_3_-induced leaf senescence could affect the population fitness of sap-sucking insects via changes in foliar nitrogenous nutrition and defense metabolism.

*Bemisia tabaci* is a sucking insect that is regarded as the most destructive agricultural invasive pest in China. *B. tabaci* causes extensive crop losses annually, estimated at billions of dollars, through feeding directly and virus transmission (Dalton, 2006). Understanding the physiological basis involved in climate change-driven outbreak of invasive insects is crucial to crop production health and security. Here, we used two tomato (*Lycopersicon esculentum*) genotypes that differed in sensitivity to ET signals to determine how ET signaling pathway regulated leaf senescence under elevated O_3_, and related bottom-up effects on *B. tabaci*. Our specific goals were to determine the differences in these two plant genotypes in (i) leaf senescence under elevated O_3_; (ii) nitrogenous nutrition and resistance; and (iii) population abundance of *B. tabaci*.

## Materials and Methods

### Treatments Under Different O_3_ Concentrations

The field experiments were carried out in eight 2.1 m diameter and 2 m height octagonal, open-top chambers (OTCs) at the Observation Station of the Global Change Biology Group, Institute of Zoology, Chinese Academy of Sciences in Xiaotangshan County, Beijing, China (40°11′N, 116°24′E). The O_3_ treatments were set up as: current tropospheric O_3_ levels (40 nL L^-1^) and elevated O_3_ levels (90 nL L^-1^). The O_3_ treatment was performed in four paired OTCs. Each OTC with elevated O_3_ was matched with one OTC with ambient O_3_. The OTCs were ventilated with air daily from 8:00 a.m. to 6:00 p.m. In the elevated O_3_ treatment, the method of O_3_ generation was offered by Cui et al. (2012). O_3_ concentrations were monitored (AQL-200, Aeroqual, New Zealand) four times per day throughout the studies to keep relatively stable O_3_ levels within the OTCs. The O_3_ levels throughout the research were 42 ± 3.8 nL L^-1^ in the ambient O_3_ OTCs and 89 ± 5.3 nL L^-1^ in the elevated O_3_ OTCs. Air temperatures were measured and there was not obviously difference between the two treatments (22.7 ± 1.9°C in ambient O_3_ chambers vs 24.2 ± 2.0°C in elevated O_3_ chambers).

### Plants and Insects

Wild-type Ailsa Craig (AC) and ET-insensitive mutation *Never ripe* (*Nr*) tomato plants were kindly supplied by Professor Chuanyou Li (Institute of Genetics and Developmental Biology, Chinese Academy of Sciences). The N-terminal coding region of an ET receptor (*Le-ETR3*/*NR*) was associated with a single base substitution in ET-insensitive mutation *Nr*. *Nr* plants showed defects in the ET-induced triple response in etiolated hypocotyls and also exhibited the lack of fruit ripening (Lanahan et al., 1994; Wilkinson et al., 1995).

The germinated seeds were individually sown into approximate 1.5-L small pots. Tomato plants were maintained in the OTCs for 43 days from seedling with two to three leaves to the end of the experiment (19 May to 30 June 2015). The position of pot within each chamber was re-randomized once every week. There were 88 tomato plants within each OTC (704 plants in total), which contained 54 AC plants and 34 *Nr* plants. The insecticides were not used throughout the research. The plants were irrigated every 2 days.

The *B. tabaci* Mediterranean genetic group, also called Q biotype, was kindly provided by Professor Youjun Zhang (Institute of Vegetables and Flowers, Chinese Academy of Agricultural Sciences). The *B. tabaci* population was maintained on the cotton plants in separated cages in a greenhouse at 25 ± 2°C and 75 ± 10% relative humidity, with a photoperiod of 14 h light: 10 h dark. The 30 adults were sampled to determine the *B. tabaci* biotypes of colony by sequencing a molecular marker *mtCO I* (mitochondrial cytochrome oxidase I) gene (De Barro et al., 2011).

### ET Precursor ACC and ET Inhibitor 1-MCP

During O_3_ exposure, 320 plants in total in eight OTCs, which contained 40 tomato plants (30 AC plants and 10 *Nr* plants) with uniform size in per OTC, were randomly selected. Ten AC plants were sprayed with ACC (Merck Millipore, Darmstadt, Germany, AC/ACC plants) and 10 AC plants were sprayed with 1-MCP (Yuanye, Beijing, China, AC/1-MCP plants). Ten AC plants and 10 *Nr* plants were sprayed with H_2_O (AC/H_2_O and *Nr*/H_2_O), which was regarded as control treatment. Both sides of leaves were sprayed once every two days at 8:00 a.m. along the 43 days of the experiment. Treatment of samples with ACC was conducted by dissolving 30 mg of ACC in 1 L of distilled water with 100 μL L-77 at a final concentration of 50 ppm. The final concentration of 1-MCP was 1 ppm by dissolving 0.005 g 1-MCP into distilled water (1 L) with 100 μL L-77 (Kevany et al., 2007). After 38-day O_3_ fumigation (19 May to 25 June 2015), the leaves from 40 tomato plants of each OTC (10 AC/H_2_O plants, 10 AC/ACC plants, 10 AC/1-MCP plants, and 10 *Nr*/H_2_O plants) were collected and immediately frozen in liquid nitrogen to analyze ROS accumulation, ET emission, and the expression of ET synthase genes.

### *Bemisia tabaci* Infestation

Plants were arranged for two different treatments with *B. tabaci*. After 21-day O_3_ fumigation (19 May to 9 June 2015), 128 tomato plants in total in eight OTCs, which contained 16 tomato plants (eight AC plants and eight *Nr* plants) with uniform size in per OTC, were randomly selected for 21-day *B. tabaci* infestation experiment. Each plant was inoculated with five pairs of newly emerging *B. tabaci*. The *B. tabaci* was maintained in a clip-cage to develop and produce offspring on tomato plants for 3 weeks. All *B. tabaci* stages (eggs, one to four nymphs and adults) on per plant were counted as the *B. tabaci* abundance in 30 June 2015.

In the second part of this experiment, 128 tomato plants in total in eight OTCs, which 16 tomato plants (eight AC plants and eight *Nr* plants) with uniform size in per OTC, were randomly selected after 41-day O_3_ fumigation (19 May to 28 June 2015). Each plant was damaged with ten pairs of newly emerging *B. tabaci*. The *B. tabaci*, which was maintained within a clip-cage, infested freely for 24 h. Another 128 tomato plants in total in eight OTCs, which included 16 tomato plants (eight AC plants and eight *Nr* plants) with uniform size in per OTC, were also randomly selected after 41-day O_3_ fumigation (19 May to 28 June 2015). These plants were not infested with *B. tabaci*, which served as un-infested control. The un-infested and 24 h-infested leaves of each tomato plants were harvested in 29 June 2015 and immediately frozen in liquid nitrogen for amino acid, N concentration, hormone, ET emission, and the expression of hormone-signal related genes analysis.

### Plant Photosynthesis and Growth Traits

Eight tomato plants of each cultivar per chambers were randomly selected for determining net photosynthetic rate by using a Li-Cor 6400 gas exchange system (LI-COR, Inc., Lincoln, NE, United States). Leaf chlorophyll content of tomato plant was measured with a Minolta SPAD-502 plus (Konica Minolta Sensing, Inc., Osaka, Japan).

### Reactive Oxygen Species (ROS) Accumulation

Frozen powder, which was hand ground in liquid nitrogen, was weighed and immediately homogenized with 10 mM Tris-HCl buffer (pH 7.3). The homogenized extract was centrifuged twice at 15,000 rpm for 5 min. The quantification of ROS was determined by 10 mM H_2_DCFDA (Aladdin, Shanghai, China), which was dissolved in DMSO, and incubated for 10 min in darkness at room temperature. Fluorescence absorbance was determined by a SpectraMax *i3* (Bio-Rad, Hercules, CA, United States). The quantification of total protein was measured by Bradford dye. The ROS production was expressed as relative fluorescence units (RFUs) per milligram of protein.

### Amino Acids and N Concentration

Approximately 0.2 g leaf samples were homogenized in liquid nitrogen, and then were extracted within 2.5 mL of cold 5% acetic acid. The extraction was agitated for 1 h on a shaker (C. Gerhardt GmbH & Co., KG, Königswinter, Germany) at room temperature. Homogenates were centrifuged at 4000 rpm for 15 min, and the supernatants were used for leaf individual amino acid analyses. The leaf amino acids were measured by reverse-phase HPLC with precolumn derivatization using *o*-phthaldialdehyde (OPA) and 9-fluorenylmethyloxycarbonyl (FMOC). The quantification of amino acids was calculated with reference to the standard curves of AA-S-17 (Agilent, Palo Alto, CA, United States, PN: 5061-3331) amino acid mixture, supplemented with asparagine, glutamine, and tryptophan (Sigma-Aldrich Co., St. Louis, MO, United States). Free amino acid concentrations of the five standard solutions were 250, 100, 50, 25, and 10 pmol μL^-1^. The mixed sample with 10 μL amino acid sample, 20 μL sodium borate buffer (0.4N, pH 10.4), 10 μL OPA, 10 μL FMOC, and 50 μL water was injected to the HPLC (Agilent Technologies, Palo Alto, CA, United States). The HPLC analysis was performed using a method provided by Guo et al. (2015). Total amino acids in leaves were measured according to a method as described previously (Chen et al., 2004). N concentration in leaves was measured using Kjeltec N analysis (Foss automated Kjeltec^TM^ instruments, Model 2100, Hillerød, Denmark).

### ET Emission

The ET emission from leaves was determined according to Wilkinson and Davies (2009) with some modification. After 15 min, the excised leaves were attached to a water-saturated filter paper in sealed vials at room temperature. The containers were flushed with fresh air from outside the laboratory for 1 min and then immediately capped with a rubber septum lid. After 1 h, 1 mL gas from the vial headspace was withdrawn with gas-tight syringes, and injected to gas chromatography (7890A, Agilent Technologies UK Ltd., Wokingham, United Kingdom) fitted with a GS-GASPRO (60 m × 0.320 mm) column. The temperature was maintained at 80°C for 4 min to resolve ET and then increased at 25°C min^-1^ to 250°C and held for 10.8 min. The flow rate of hydrogen carrier gas was 40 mL min^-1^ and was detected by flame ionization detector (FID). The ET production rate was estimated by comparison of sample peak areas of known ET standards (BOC Special Gases, Manchester, United Kingdom) and corrected for tissue fresh weight and the duration of incubation to determine ET emission rate.

### Hormone Analysis

The foliar hormone was measured according to Guo et al. (2017) with some modification. Approximately 300 mg of plant tissue was hand ground in liquid nitrogen and was quickly homogenized in 0.5 mL extraction buffer for 30 min at 4°C with gentle agitation on a shaker. Subsequently, each sample was additionally added 1 mL of CH_2_Cl_2_, and then agitated for 30 min on a shaker at 4°C. The homogenized sample was centrifuged at 13,000 *g* for 10 min. After centrifugation, the lower layer was collected, and then was concentrated in a dry machine. The concentrated sample was re-solubilized in 200 μL of MeOH. Next, 1 μL of the sample was injected into an Agilent ZORBAX SB-Aq column (600 bar, 2.1 mm × 100 mm, 1.8 *μ*m) for hormone analysis. The hormone contents were calculated with reference to standard curves.

### Protease Activity

Approximately 200 mg tomato leaf samples were ground in liquid nitrogen. It was mixed with 1 mL 50 mM Tris-HCL buffer (pH 7.5) at 4°C for 30 min. The homogenized extract was centrifuged at 4°C, 12,000 rpm for 40 min. After centrifugation, the upper layer was collected for protease activity analysis. The supernatants (100 μL) was mixed with 600 μL 50 mM pH 8.0 Na-Pi buffer (50 mM pH 5.0 citric acid- phosphate buffer; pH 7.5 Na-Pi; pH 9.5 Tris-HCL buffer; pH 11 NaOH-NaHCO_3_) containing 100 μL 0.6% (w/v) azocasein (Sigma, United States), and the mixture was incubated at 37°C for 3 h. The reaction was terminated by adding 400 μL 10% TCA (Aladdin, Shanghai, China). The mixture was maintained at 4°C for 30 min and then centrifuged at 4°C, 10,000 rpm for 10 min. The absorbance of the filtrate at 366 nm was determined by fluorescence spectrophotometry (SpectraMax *i3*, Molecular Devices, United States; Wang et al., 2004).

### RNA Extraction and Quantitative PCR (qPCR) Analysis

Gene expression was measured using quantitative reverse transcription polymerase chain reaction. Each treatment was replicated with four biological repeats and four technical repeats. The RNA easy Mini Kit (Qiagen) was used to isolate total RNA from the leaves. The cDNA was generated from 1 *μ*g of RNA. We used real-time quantitative PCR (qPCR) to determine the mRNA levels. Specific primers for each gene were designed from The EST sequences was used to design specific primers for target genes using PRIMER5 software (**Table 1**). The qPCR reactions were performed in a 20 *μ*L total reaction volume that included 10 *μ*L of 2× SYBR Premix EX Taq^TM^ (Qiagen) master mix, 5 mM of each gene-specific primer, and 1 *μ*L of pure cDNA template. Reactions were carried out using the Mx 3000P detection system (Stratagene), with the parameters [the elongation temperature (68°C)] as described in Guo et al. (2015). According to the studies about reference genes used in tomato plants (Expósito-Rodríguez et al., 2008; Løvdal and Lillo, 2009; Mascia et al., 2010), we chose five different reference genes including *ACTIN, EF-1, TIPL-41, GADPH*, and *TUB*, to get the best reference gene, which is expressed at a relative constant level among different experimental treatments, in my experimental conditions. We used *TIP41* and *Actin* as the internal qPCR standard. The expression level of each target gene was standardized to the tomato *TIP41* gene and *Actin* gene (Expósito-Rodríguez et al., 2008).

**Table 1:** Primer sequences used for real-time quantitative PCR.

Gene	GenBank	Primer sequence(5′-3′)
	accession no.	
*PR* (*pathogenesis-related protein*)	Solyc01g106620.2	*F*: GAGGGCAGCCGTGCAA *R*: CACATTTTTCCACCAACACATTG
*GLU* (*beta-1, 3-glucanase*)	CK664757	*F*: GCGGTGTTCAGCCTGGATG *R*: AGCATGAGCAAGAAGTATGTTGTG
*LOX* (*lipoxygenase*)	U37840	*F*: GACTGGTCCAAGTTCACGATCC *R*: ATGTGCTGCCAATATAAATGGTTCC
*PI* (*proteinase inhibitor*)	K03291	F: GAAAATCGTTAATTTATCCCACCG R: ACATACAAACTTTCCATCTTTACCA
*ACO* (*1-aminocyclopropane-1-carboxylate oxidase*)	X58273	*F*: GCCAAAGAGCCAAGATTTGA *R*: TTTTTAATTGAATTGGGATCTAAGC
*ACS* (*1-aminocyclopropane-1-carboxylate synthase*)	X59139	*F*: GGTCTTGCTGAAAATCAGCTTTGT *R*: AGTTGGCAATGGCCTTGAATG
*ERF1 (ethylene-responsive factor 1)*	AY044236	*F*: AGAGACCAAGGACCCCTCAT *R*: AGTAGAGACCAAGGACCCCTC
*ERF2* (*ethylene-responsive factor 2*)	AY192368	*F*: AAGGGGTTAGGGTTTGGTTAGG *R*: CAAGCAATGTTCAAGGGAGGG
*TIP41*	SGN-U321250	*F*: AGGCCTTGTCTTCGAAAGGA *R*: TCCTTTAGGACACTCCAACATGG
*Actin*	AB199316	*F*: TGGTCGGAATGGGACAGAAG *R*: CTCAGTCAGGAGAACAGGGT

### Statistical Analyses

The statistical package IBM SPSS Statistics 21.0 was used for statistical analyses. A split–split plot design was applied to analyze the hormone content and gene expression of defense signaling, which the main factor was O_3_ and block (a pair of OTCs with elevated and ambient OTCs), the subplot factor was tomato genotypes, and the sub-subplot factor was *B. tabaci* infestation. The main effects of O_3_ concentrations, tomato genotype, and *B. tabaci* infestation on plant were tested according to the following model:

$$\begin{array}{c} X_{ijklm}\;=\;\mu \;+\;O_{i}\;+\;B{\left(O\right)}_{j(i)}\;+\;G_{k}\;+\;OG_{ik}\;+\;GB{\left(O\right)}_{kj(i)}\;+\;W_{l}\;+\;OW_{il}\;+\;WB{\left(O\right)}_{lj(i)}+\;GWB{\left(O\right)}_{klj(i)}\;+\;e_{m(ijkl)} \end{array}$$

where *O* is the O_3_ treatment (*i* = 2), *B* is the block (*j* = 4), *G* is the tomato genotype (*k* = 2), and *W* is the *B. tabaci* infestation (*l* = 2). X*_ijklm_* represents the error because of the smaller scale differences between samples and variability within blocks (SPSS 21.0, SPSS Inc., Chicago, IL, United States). Effects were considered significant if *P* < 0.05. Tukey’s multiple range tests were used to separate means when ANOVAs were significant (*P* < 0.05). The ET emission, biomass, photosynthetic rate, chlorophyll content, ROS, curl leaves, O_3_-damaged stippled leaves, deciduous leaves of plants, population abundance, individual amino acids, and total amino acid under two O_3_ concentrations were analyzed by a split-plot design, which O_3_ and block as the main effects and tomato genotype as the subplot effect.

## Results

### Elevated O_3_ Increased the ET Synthesis and Emission of Tomato Plants

**FIGURE 1:**
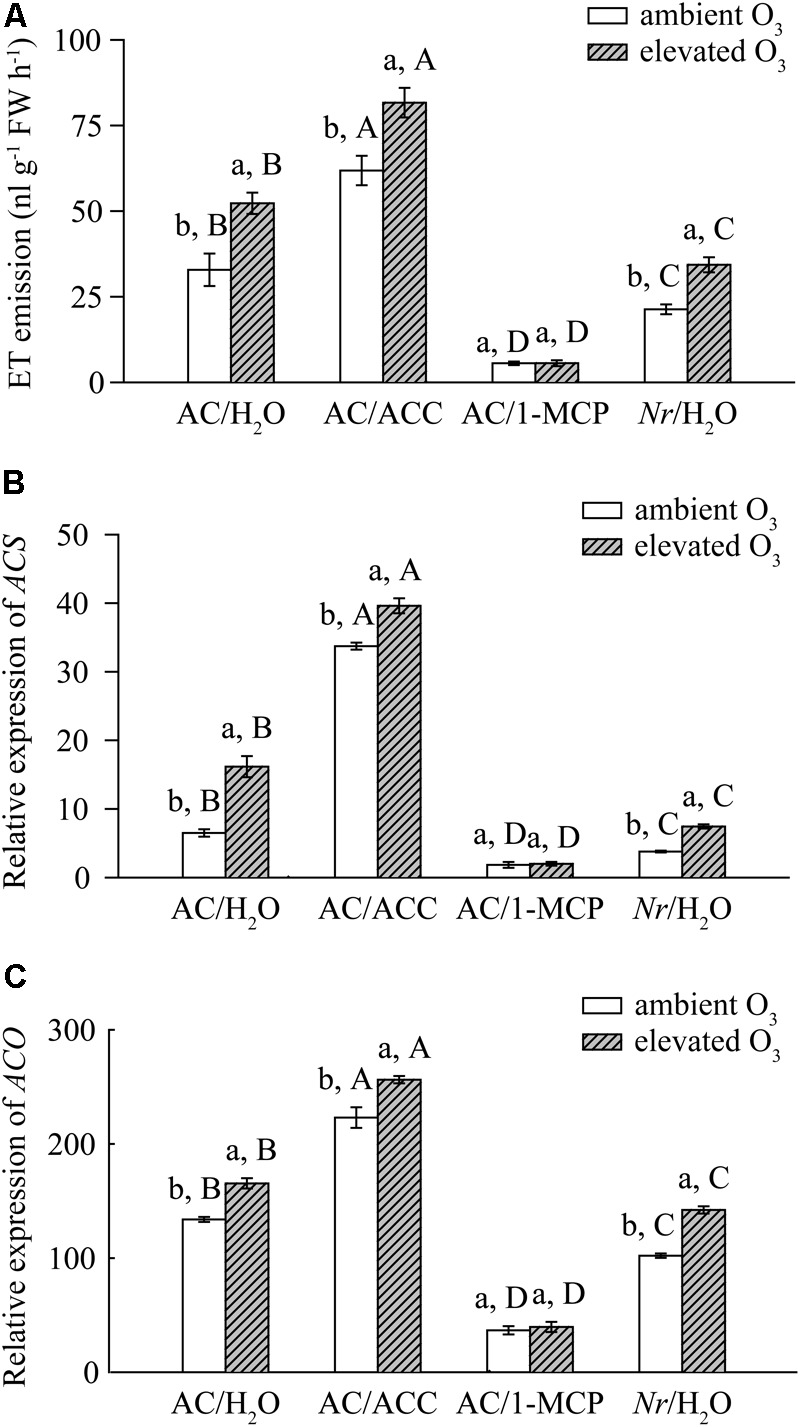
The ET production rate and fold-change in the expression of ET synthesis genes of wild-type AC plants spraying with ACC, 1-MCP, and H_2_O, and of ET-insensitive *Nr* mutants spraying with H_2_O grown under ambient O_3_ and elevated O_3_. **(A)** ET production rate. **(B)** *ACS*. **(C)** *ACO*. Each value represents the mean (±SE) of four OTCs (10 plants for each treatment per OTC). Different lowercase letters indicate significant differences between ambient O_3_ and elevated O_3_ within the same genotype. Different uppercase letters indicate significant differences between genotypes following with ACC, 1-MCP, or H_2_O applications within the same O_3_ treatment.

Elevated O_3_ significantly increased the ET production by 37% in AC plants spraying with H_2_O (AC/H_2_O plants), by 24% in AC plant spraying with ACC (AC/ACC plants), and by 38% in *Nr* plants spraying with H_2_O (*Nr*/H_2_O plants). However, ET production was not affected by elevated O_3_ in AC plants spraying with 1-MCP (AC/1-MCP plants). Under both O_3_ concentrations, the ET production was the highest in AC/ACC plants and the lowest in AC/1-MCP plants. The level of ET emission was significantly higher in AC/H_2_O plants than in *Nr*/H_2_O plants (**Figure 1A**). We also analyzed *ACS* and *ACO* genes, which were two important ET synthesis genes in tomato plants. The expression of foliar *ACO* and *ACS* genes were consistent with the level of ET emission, which were upregulated under elevated O_3_ in AC/H_2_O plants, AC/ACC plants, and *Nr*/H_2_O plants (**Figures 1B,C**).

### O_3_-Induced Leaf Senescence Was Dependent on ET Signaling Pathway

**FIGURE 2:**
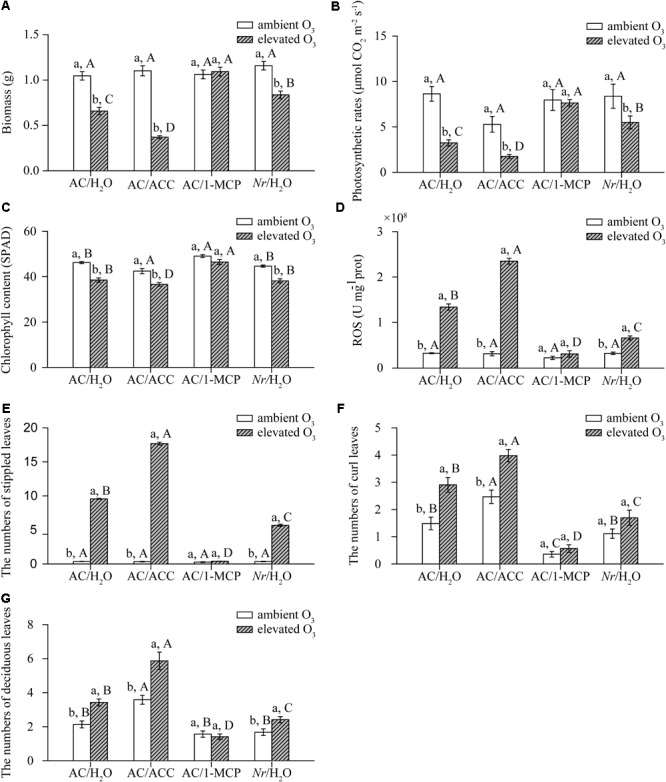
The phenotype of wild-type AC plants spraying with ACC, 1-MCP, and H_2_O, and of ET-insensitive *Nr* mutants spraying with H_2_O grown under ambient O_3_ and elevated O_3_. **(A)** Biomass. **(B)** Photosynthetic rate. **(C)** Chlorophyll content. **(D)** ROS. **(E)** O_3_-damaged stippled leaves. **(F)** Curl leaves. **(G)** Deciduous leaves. Each value represents the mean (±SE) of four OTCs (10 plants for each treatment per OTC). Different lowercase letters indicate significant differences between ambient O_3_ and elevated O_3_ within the same genotype. Different uppercase letters indicate significant differences between genotypes following with ACC, 1-MCP, or H_2_O applications within the same O_3_ treatment.

Elevated O_3_ decreased the plant biomass by 37%, the photosynthetic rate by 62%, and chlorophyll content by 17% in AC/H_2_O plants. The plant biomass, photosynthetic rate, and chlorophyll content were not affected by elevated O_3_ in AC/1-MCP plants. Elevated O_3_ had the most detrimental effects on AC/ACC plants, reducing plant biomass by 66%, photosynthetic rate by 67%, and chlorophyll content by 14%. Compared with AC/H_2_O plant, elevated O_3_ had marginal effects on *Nr*/H_2_O plants, with decreased biomass of 27%, photosynthetic rate by 34%, and chlorophyll content by 15% (**Figures 2A–C**).

Elevated O_3_ also increased ROS accumulation and leaf injury, including numbers of O_3_-damaged stippled leaves, deciduous leaves, and curl leaves in AC/H_2_O plants, AC/ACC plants, and *Nr*/H_2_O plants. ROS accumulation and leaf injury in AC/H_2_O plants were significantly lower than these in AC/ACC plants, and higher than these in *Nr*/H_2_O plants. In AC/1-MCP plants, ROS accumulation and leaf injury were similar under both O_3_ concentrations (**Figures 2D–G**).

### Elevated O_3_ Increased the Population Abundance of *B. tabaci* on Tomato Plants

**FIGURE 3:**
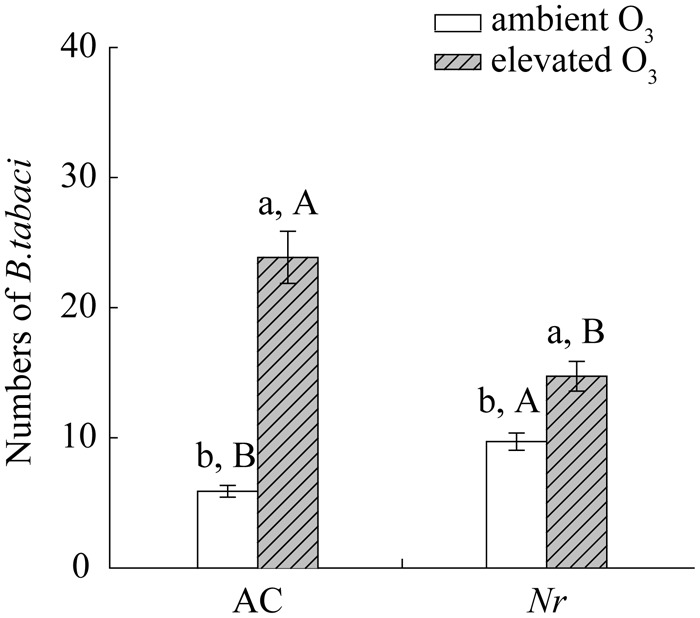
Abundance of *B. tabaci* when fed on two tomato genotypes grown under ambient O_3_ and elevated O_3_. Each value represents the mean (±SE) of four OTCs (eight plants for each genotype per OTC). Different lowercase letters indicate significant differences between ambient O_3_ and elevated O_3_ within the same genotype. Different uppercase letters indicate significant differences between genotypes within the same O_3_ treatment.

Ozone concentration and plant genotype had significant effects on the population abundance of *B. tabaci*. Relative to ambient O_3_, the number of *B. tabaci* was increased 3-fold on AC plants and 0.5-fold on *Nr* plants under elevated O_3_ (**Figure 3**).

O_3_-induced leaf senescence activated leaf salicylic acid (SA) and ET signaling pathway, but had no effects on jasmonic acid (JA) signaling pathway.

**FIGURE 4:**
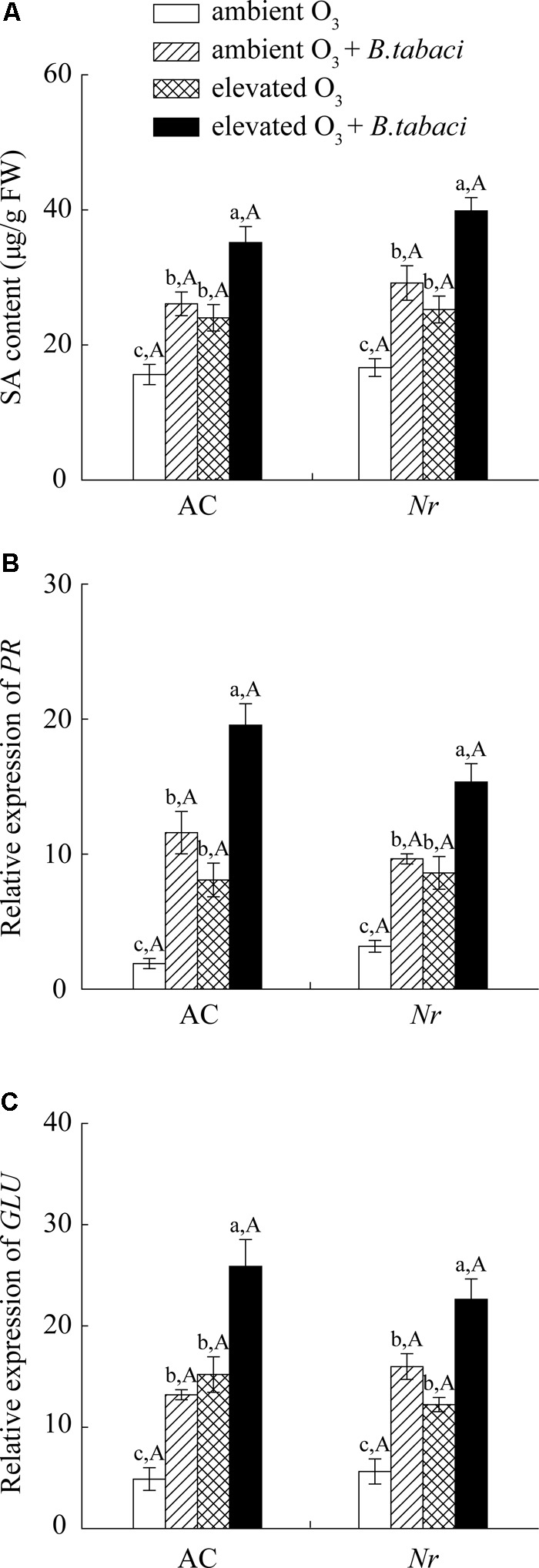
SA content and fold-change in the expression of related genes involved in the SA-dependent signaling pathway in two tomato genotypes grown under ambient O_3_ and elevated O_3_ with and without *B. tabaci* infestation. **(A)** SA content. **(B)** *PR*. **(C)** *GLU*. Each value represents the mean (±SE) of four OTCs (eight plants for each genotype per OTC). Different lowercase letters indicate significant differences among the combinations of *B. tabaci* treatment and O_3_ concentrations within the same genotype. Different uppercase letters indicate significant differences between genotypes within the same O_3_ treatment and *B. tabaci* treatment.

Ozone concentration and *B. tabaci* infestation had significant effects on the foliar SA content. Regardless of *B. tabaci* infestation, elevated O_3_ significantly increased foliar SA content by 54% in AC plants and by 51% in *Nr* plants (**Figure 4A**). Under both O_3_ levels, *B. tabaci* infestation increased the foliar SA content in AC and *Nr* plants. Regardless of *B. tabaci* infestation and O_3_ concentration, foliar SA content was equivalent in AC and *Nr* plants. The expression of β*-1,3-glucanase* (*GLU*) and *pathogenesis-related protein* (*PR*) genes, two downstream genes of SA signaling pathway, was consistent with foliar SA content, which was increased under elevated O_3_ and *B. tabaci* infestation, and was not affected by plant genotype (**Figures 4B,C**).

**FIGURE 5:**
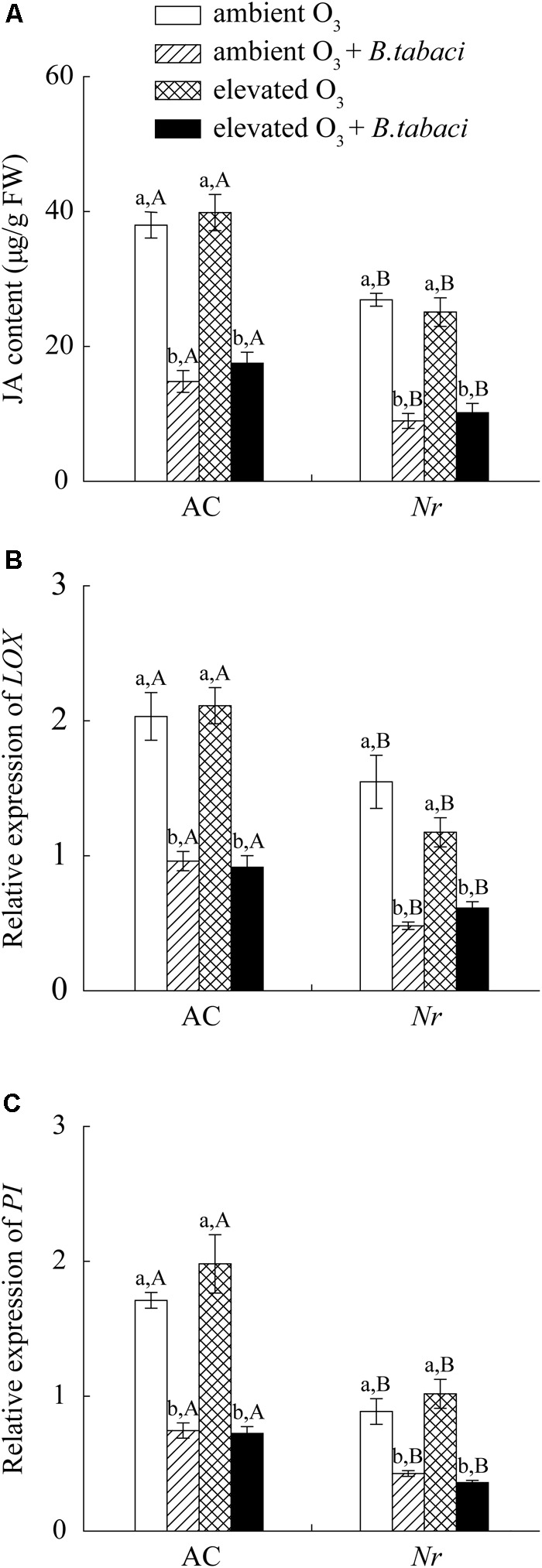
JA content and fold-change in the expression of related genes involved in the JA-dependent signaling pathway in two tomato genotypes grown under ambient O_3_ and elevated O_3_ with and without *B. tabaci* infestation. **(A)** JA content. **(B)** *LOX.* **(C)** *PI*. Each value represents the mean (±SE) of four OTCs (eight plants for each genotype per OTC). Different lowercase letters indicate significant differences among the combinations of *B. tabaci* treatment and O_3_ concentrations within the same genotype. Different uppercase letters indicate significant differences between genotypes within the same O_3_ treatment and *B. tabaci* treatment.

Elevated O_3_ did not increase the foliar JA accumulation and the relative expression level of *lipoxygenase* (*LOX*) and *proteinase inhibitor* (*PI*), which were two marker genes of JA signaling pathway, in AC and *Nr* plants with and without *B. tabaci* infestation. Under both O_3_ concentrations, *B. tabaci* infestation significantly reduced the foliar JA concentration and the relative expression level of *LOX* and *PI*. Regardless of *B. tabaci* infestation and O_3_ concentration, the foliar JA concentration and the expression of *LOX* and *PI* were markedly higher in AC plants than in *Nr* plants (**Figure 5**).

**FIGURE 6:**
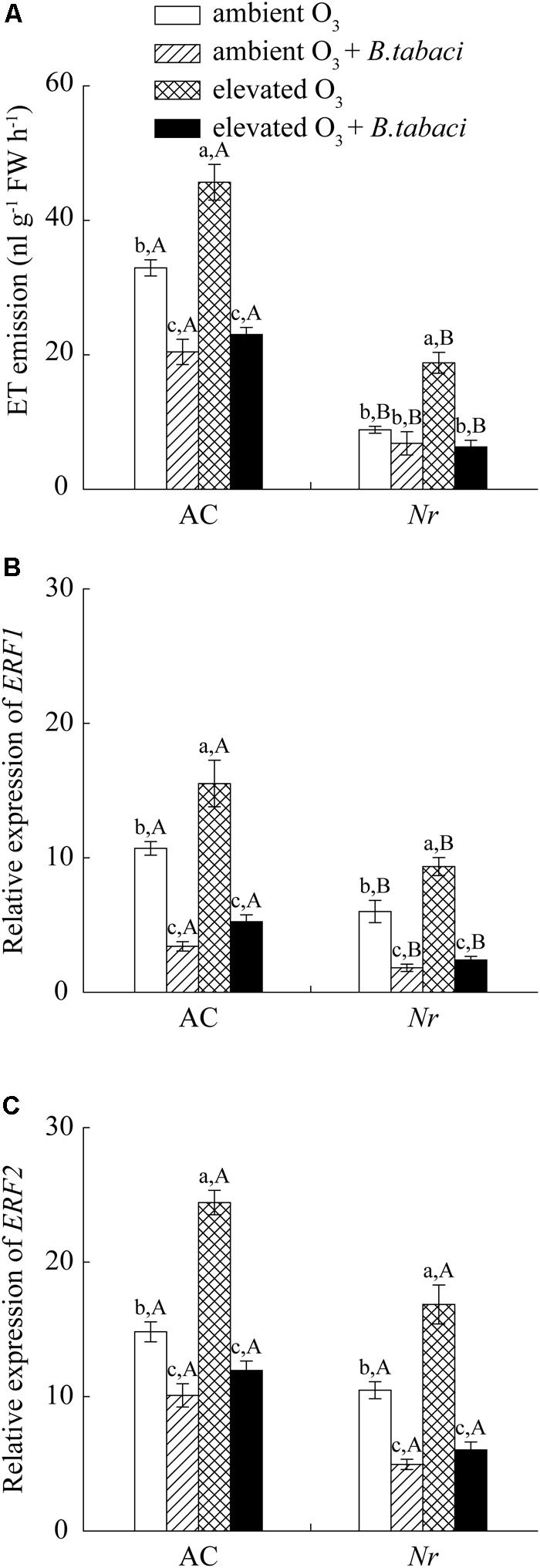
Ethylene emission and fold-change in the expression of related genes involved in the ET-dependent signaling pathway in two tomato genotypes grown under ambient O_3_ and elevated O_3_ with and without *B. tabaci* infestation. **(A)** ET emission. **(B)** *ERF1*. **(C)** *ERF2*. Each value represents the mean (±SE) of four OTCs (eight plants for each genotype per OTC). Different lowercase letters indicate significant differences among the combinations of *B. tabaci* treatment and O_3_ concentrations within the same genotype. Different uppercase letters indicate significant differences between genotypes within the same O_3_ treatment and *B. tabaci* treatment.

Elevated O_3_ significantly increased the emission of ET without *B. tabaci* infestation in AC plants, increasing from 33 to 44 nL g^-1^FW h^-1^. When infested by *B. tabaci*, elevated O_3_ had no effects on the emission of ET. *B. tabaci* infestation significantly decreased the ET emission by 38 % under ambient O_3_, and by 49% under elevated O_3_ in AC plants. Compared with AC plants, the ET emission was significantly lower in *Nr* plants regardless of *B. tabaci* infestation and O_3_ concentrations (**Figure 6A**). We also analyzed the *ethylene-response factor 1* (*ERF1*) and *ethylene-response factor 2* (*ERF2*), two down-stream genes of ET signaling pathway, and found that their expression was increased by elevated O_3_, and decreased by *B. tabaci* infestation in both tomato genotypes (**Figures 6B,C**).

### O_3_-Induced Leaf Senescence Improved the N Nutrition of Tomato Plants

**FIGURE 7:**
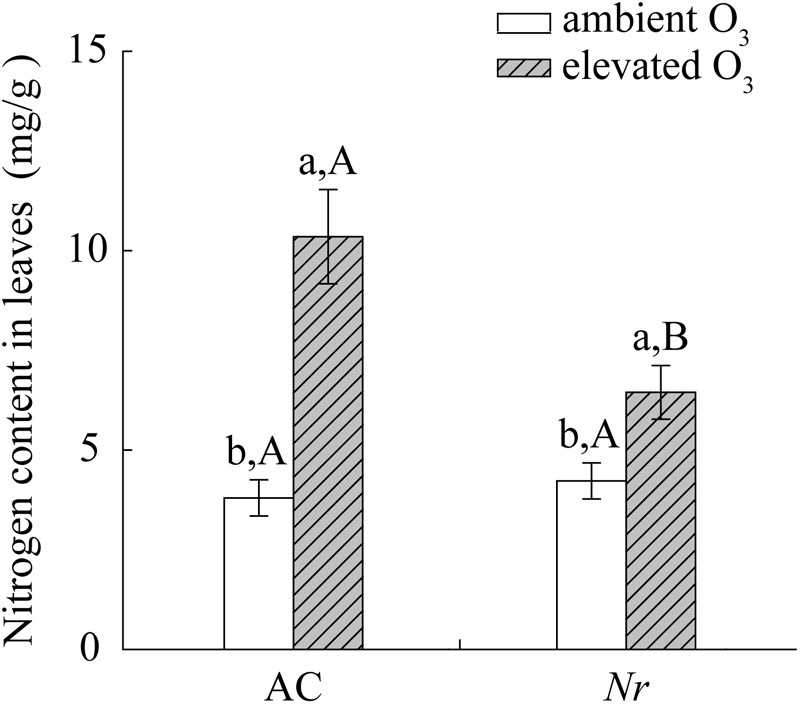
Total nitrogen concentration for two tomato genotypes grown under ambient O_3_ and elevated O_3_ without *B. tabaci* infestation. Different lowercase letters indicate significant differences between ambient O_3_ and elevated O_3_ within the same genotype. Different uppercase letters indicate significant differences between genotypes within the same O_3_ treatment.

**FIGURE 8:**
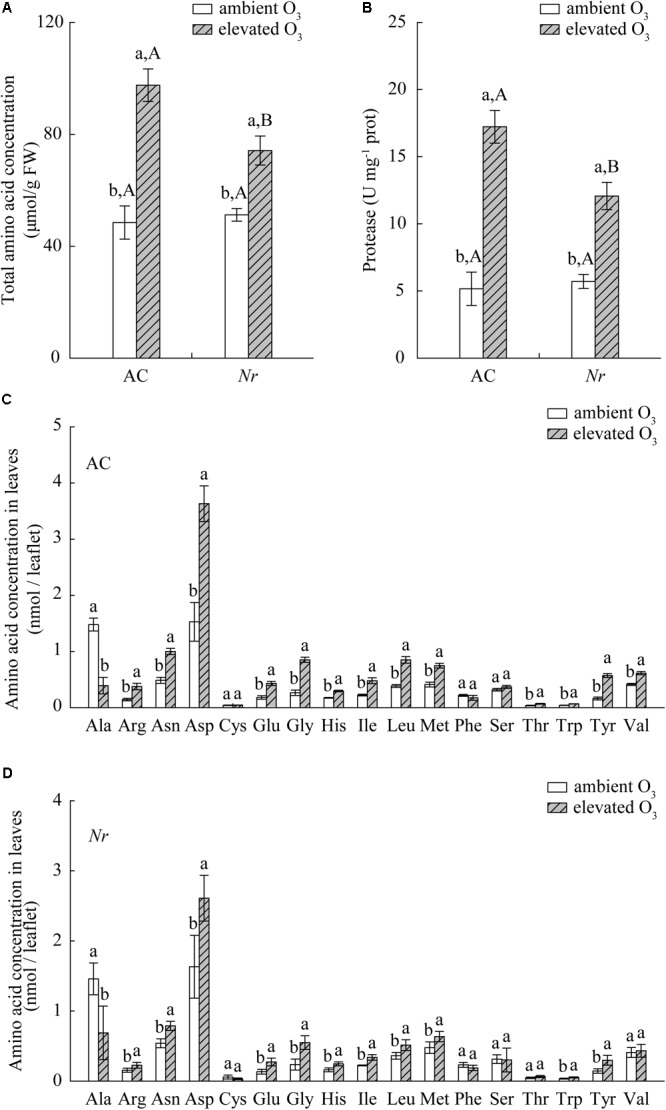
Total and individual amino acid concentration for two tomato genotypes grown under ambient O_3_ and elevated O_3_ without *B. tabaci* infestation. **(A)** Total amino acid concentration. **(B)** The activity of protease. **(C)** Individual amino acid concentration in AC plants. **(D)** Individual amino acid concentration in *Nr* plants. Different lowercase letters indicate significant differences at *P* < 0.05.

Elevated O_3_ significantly increased foliar nitrogen content, total amino acid content, and protease activity in AC and *Nr* plants, but the response was greater in AC plants (1.6-fold, 2.7-fold, and 2-fold) compared with *Nr* plants (1.3-fold, 1.5-fold, and 1.4-fold; **Figures 7, 8A,B**).

A total of 17 individual amino acids in leaves were analyzed including essential and non-essential amino acids. Elevated O_3_ markedly increased the concentrations of 13 individual amino acids, including nine essential amino acids (Arg, His, Ile, Leu, Met, Thr, Trp, Tyr, and Val) and four non-essential amino acids (Asn, Asp, Glu, and Gly) in AC/H_2_O plants, and 11 individual amino acids including six essential amino acids (Arg, His, Ile, Leu, Met, Trp, and Tyr) and four non-essential amino acids (Asn, Asp, Glu, and Gly) in *Nr*/H_2_O plants. Furthermore, the increase in individual amino acids was greater in AC plants than in *Nr* plants (**Figures 8C,D**).

## Discussion

Hormone-dependent signals can act both independently and interactively to modulate the plant response to climate change (elevated CO_2_ or O_3_) and insect infestation (Baier et al., 2005; Tamaoki, 2008; Erb et al., 2012; Zavala et al., 2013; Pellegrini et al., 2016). The hormone-mediated changes in host plant phenotypes under climate change can further affect the performance of herbivorous insects (Guo et al., 2014, 2017). Here, we report that O_3_, as a strong oxidative stressor, activates ET signaling pathway, which is involved in mediating O_3_-induced leaf senescence. Furthermore, leaf senescence under elevated O_3_ is associated with changes in plant quality, which has no effects on hormone-dependent defense but increases amino acid concentrations, and therefore increases the number of *B. tabaci* on wild-type plants. Compared with wild-type plants, O_3_-induced leaf senescence is mitigated in *Nr* plants, which dramatically reduces the beneficial effects of O_3_-induced leaf senescence on *B. tabaci*. Consequently, although ET signaling pathway is important in improving plant resistance to insect infestation under non-stress conditions (Louis et al., 2015), our results demonstrate that O_3_-induced stimulation of ET signaling pathway in plant that accelerates leaf senescence boosts *B. tabaci* infestation.

Ethylene emission is one of the most quickly responses to O_3_ exposure in host plants (Moeder et al., 2002; Gupta et al., 2005), and is correlated with plant sensitivity to O_3_ stress (Pellegrini et al., 2013; Vainonen and Kangasjärvi, 2015). Plants with mutation in ET signaling pathway are less sensitive to O_3_ exposure, and plants with ET overproduction are more sensitive to O_3_ exposure (Tamaoki et al., 2003). Our results also found that ET-overproducing AC/ACC plants were more sensitive, and ET insensitive AC/1-MCP plants were not sensitive to O_3_ exposure than wild-type AC/H_2_O plants. However, in contrast to an earlier study, ET-insensitive *Nr* plants exhibit a similar degree of O_3_-induced leaf lesions with wild-type Pearson plants under acute O_3_ fumigation with 200 ppb for 4 h (Castagna et al., 2007). In the current study, *Nr* plants exhibited lower tissue injury, lower ROS accumulation, and grew better than AC plants under chronic O_3_ exposure with 89 ppb for 21 days (**Figure 2**). It is likely that different fumigation regimes of O_3_ exposure may be important for the different function of *NR* receptor in regulating O_3_ sensitivity. Acute O_3_ exposure means that plants are fumigated with O_3_ concentration exceeding 120 ppb within a few hours, while chronic O_3_ exposure is daily peak concentration in the range of 40–120 ppb within several days (Long and Naidu, 2002). Many works suggest that acute and chronic O_3_ exposure induce different mechanisms (Kollist et al., 2007; Wittig et al., 2007; Chen et al., 2009). In soybean plants, chlorophyll fluorescence image indicates that acute O_3_ exposure causes small area reduction in photosynthetic capacity near the major vein by direct oxidative damage to PSII. Chronic O_3_ exposure depresses photosynthetic capacity around interveinal regions through affecting Rubisco (Chen et al., 2009). Stomatal movement is also different under acute and chronic O_3_ exposure. Acute O_3_ exposure induces a rapid stomatal closure and then recovers to an original rate of stomatal conductivity (Kollist et al., 2007). Chronic O_3_ exposure causes a continuous decline of stomatal conductivity (Kitao et al., 2009). Therefore, irrespective of acute O_3_ exposure, *NR* receptor is important for mediating plant sensitivity to chronic O_3_ exposure.

Ethylene signaling pathway can also regulate O_3_-induced cell death via cross-talking with other hormone signaling pathway, such as SA and JA signaling pathway. Early studies demonstrate that both ET and SA signaling pathways are activated in *Arabidopsis* (*Arabidopsis thaliana*) under acute O_3_ exposure (Moeder et al., 2002; Rao et al., 2002; Vahala et al., 2003). Furthermore, SA signaling pathway is requirement for O_3_-induced ET synthase, which regulates plant response to O_3_ exposure. In *Arabidopsis* double mutants crossing ET over-production *eto3* with SA-deficient *NahG* plants, O_3_-induced ET emission and necrotic lesion are obviously reduced compared with these detected in *eto3* mutants with O_3_ hyper-sensitive (Rao et al., 2002). In the current study, we also found that ET emission, SA content, ET-dependent *ERF*, and SA-dependent *PR* mRNA transcripts were significantly increased in tomato plants under chronic O_3_ exposure. In contrast to SA signaling pathway, JA signaling pathway is differently affected by acute and chronic O_3_ exposure. Acute O_3_ exposure initiates the JA signaling pathway, which is involved in regulating O_3_-induced cell death (Koch et al., 2000; Rao et al., 2000; Tuominen et al., 2004). Furthermore, experiments of JA-insensitive *jasmonate resistant 1* and methyl jasmonate pretreatment demonstrate that JA inhibits the propagation of cell death via suppressing the SA and ET signaling pathway under acute O_3_ exposure (Rao et al., 2000; Tuominen et al., 2004). However, for chronic O_3_ exposure, our results were consistent with Cui et al. (2012), which had no effects on JA content and JA-synthase *LOX* and JA-dependent *PI* mRNA transcripts.

A direct role of ET signaling pathway in regulating abiotic stress-induced leaf premature senescence has been demonstrated. Experiments in maize show that a deficiency in the ET synthase inhibits drought-induced senescence, and the delayed drought-induced senescence in ET synthase mutants is complemented by spraying with ET precursor ACC (Young et al., 2004). Similar to drought-induced leaf senescence, the acceleration of leaf senescence under O_3_ exposure has indeed been correlated with enhanced ET production in beech trees (Nunn et al., 2005). We also found that ET insensitive AC/1-MCP and *Nr*/H_2_O plants delayed O_3_-induced leaf senescence and ET-overproducing AC/ACC plants exacerbated O_3_-induced leaf senescence (**Figure 2**). ET signal also involves secondary symplastic ROS accumulation in O_3_-exposure tomato plants, in which plant spraying with ET inhibitors accumulates less H_2_O_2_ under elevated O_3_ (Moeder et al., 2002). ROS can serve as a signal molecule to accelerate leaf senescence (Jing et al., 2008). For example, the *Arabidopsis A-Fifteen* (*AAF*) gene, the *A. thaliana* ortholog of sweet potato senescence-associated gene-*SPA15*, is involved in balancing the ROS homeostasis to regulate the age and dark-induced leaf senescence, in which leaf senescence is suppressed in *aaf* T-DNA insertion mutant and promoted in *AAF* over-expression plants (Chen et al., 2011). The regulation of *AAF* in leaf senescence is dependent on *ethylene insensitive 2* (*EIN2*; Chen et al., 2011), which is an important positive regulator in ET signaling pathway (Wen et al., 2012). A recent study also shows that application of ET inhibitor 1-MCP inhibits ROS accumulation, and thus delays leaf senescence in soybean plants under high temperature stress (Djanaguiraman et al., 2011). It is accordance with current results that low ROS accumulation and alleviated leaf senescence in ET-insensitive AC/1-MCP and *Nr*/H_2_O plants (**Figure 2**). Thus, these suggest that ET signaling pathway is required in ROS accumulation and leaf senescence under elevated O_3_.

Leaf senescence, which changes plant nutrition and defense metabolisms, could be utilized by plant to regulate insect growth. There are two hypotheses to explain the effects of senescing leaves with the sign of yellowing on herbivores: (i) handicap signal hypothesis and (ii) nutrient re-translocation hypothesis. Hamilton and Brown (2001) propose the handicap signal hypothesis that senescing leaves with bright colors are detected as a warning signal of defensive commitment against autumn colonizing insect pests (Hamilton and Brown, 2001), which explains a strong preference of aphids to green leaves (Archetti and Leather, 2005). JA-dependent defense is important for regulating plant against *B. tabaci* infestation (Zarate et al., 2007; Zhang et al., 2012; Li et al., 2014). However, *B. tabaci* infestation can suppress the effective JA-dependent defense via activating SA-dependent defense (Zhang et al., 2013). When infested with *B. tabaci*, the expression of JA-dependent *VSP1* gene is decreased in *Arabidopsis* Col-0 plants, while is increased in *Arabidopsis* SA-deficient *NahG* and *npr1* plants (Zhang et al., 2013). In the current study, *B. tabaci* infestation also significantly activated SA-dependent defense, but suppressed the JA and ET-dependent defense in tomato plants regardless of O_3_ concentrations. These results suggested that elevated O_3_ had little effect on phytohormone-dependent defensive responses to *B. tabaci* infestation. It is in accordance with those studies concerning the effect of abiotic stress on plant resistance against aphid infestation (O’Neill et al., 2010; Foyer et al., 2016; Pineda et al., 2016). For example, the accumulation of secondary defensive metabolites, i.e., glucosinolate, which is induced by *Brevicoryne brassicae*, was unaffected by different water status conditions in broccoli (*Brassica oleracea*) plants (Khan et al., 2011). Thus, it seems that our results are not supported by “handicap signal hypothesis.” Our data are consistent with the “nutrient re-translocation hypothesis,” that is, senescing leaves provide a better quality of nitrogenous food for sap-sucking insects (Holopainen and Peltonen, 2002; Holopainen et al., 2009), explaining higher number of aphids on senescing autumn leaves in *B. pendula* (Holopainen et al., 2009). It is widely accepted that leaf senescence causes the degradation of N storage proteins, which releases abundant free amino acids in leaves (Lim et al., 2007). Total amino acid contents increase in early senescing leaves of *Prunus padus* (Sandström, 2000). In *Arabidopsis*, the individual amino acid content, such as Leu, Ile, Tyr, and Arg, also increases during developmental leaf senescence (Diaz et al., 2005). For sap-sucking insects, N availability in host plants, especially amino acids, is positively correlated with sap-sucking insect development (Ponder et al., 2000; Nowak and Komo, 2010). Plants with higher amino acid content sustain more *B. tabaci* eggs and attract more *B. tabaci* for feeding (Crafts-Brandner, 2002). O_3_-induced leaf senescence improved individual and total amino acids in wild-type plants (**Figure 8**), which increased the population abundance of *B. tabaci* (**Figure 3**). Compared with wild-type plants, the lower amino acid content sustained lower population number of *B. tabaci* on *Nr* plants under elevated O_3_. Thus, these results indicate that the rise in leaf amino acid concentrations, which is caused by leaf senescence, is an important aspect of the improved population fitness of *B. tabaci* under elevated O_3_.

The population abundance of Q biotypes of *B. tabaci* was increased when fed foliage grown under elevated O_3_ in the current research. This finding coincides with early results showing that elevated O_3_ increases the population fitness of aphids (Holopainen and Kainulainen, 1997; Percy et al., 2002). However, this is in contrast with a previous study of tomato–*B. tabaci* interactions, in which the population fitness of B biotype of *B. tabaci* is reduced under elevated O_3_ (Cui et al., 2012). One possible explanation is that N levels of two genotypes of tomato plants are different under elevated O_3_, i.e., increased in AC plants but decreased in CM (Castlemart) plants. This is in agreement with previous reports of multiple variations in nutrient responses to O_3_ exposure existing among plant genotypes and/or species (Couture et al., 2014). Another explanation is that the response of *B. tabaci* to elevated O_3_ is dependent on the biotypes. Previous studies also support that Q biotype exhibits higher population fitness than B biotype, such as better feeding efficiency, greater reproductive ability, shorter development time, and greater tolerance to heat stress (Mahadav et al., 2009; Liu et al., 2013). Thus, our study suggests that elevated O_3_ may exacerbate intra-species competitions between different biotypes of *B. tabaci*.

## Conclusion

Elevated O_3_ activates ET signaling pathway, which accelerates leaf senescence associated with decrease in biomass, photosynthesis, and increase in numbers of yellow leaves; however, the performance of *B. tabaci* on tomato plants is improved by increasing nitrogenous nutrition of O_3_-induced senescing leaves. This study has generated several significant findings. First, oxidative stress can accelerate leaf senescence via regulating endogenous ET signals. Second, our results support the “nutrient re-translocation hypothesis” that O_3_-induced senescing leaves with higher amino acid contents enhance the population fitness of *B. tabaci*. Finally, such changes suggest that tomato plants may suffer greater damage due to the interacting stress of direct O_3_-damage and additive infested by *B. tabaci* if tropospheric O_3_ levels increase continuously.

## Author Contributions

HG, YS, and FG planned and designed the research. HG performed the experiments, conducted the fieldwork, and analyzed the data. CL provided tomato seeds. HY provided field support. HG wrote the first draft of the manuscript. YS and FG contributed to the subsequent manuscript development.

## Conflict of Interest Statement

The authors declare that the research was conducted in the absence of any commercial or financial relationships that could be construed as a potential conflict of interest.
