# Corrigendum: O_3_-Induced Leaf Senescence in Tomato Plants Is Ethylene Signaling-Dependent and Enhances the Population Abundance of *Bemisia tabaci*

**DOI:** 10.3389/fpls.2018.01080

**Published:** 2018-07-19

**Authors:** Honggang Guo, Yucheng Sun, Hongyu Yan, Chuanyou Li, Feng Ge

**Affiliations:** ^1^State Key Laboratory of Integrated Management of Pest Insects and Rodents, Institute of Zoology, Chinese Academy of Sciences, Beijing, China; ^2^College of Life Sciences, University of Chinese Academy of Sciences, Beijing, China; ^3^State Key Laboratory of Plant Genomics, National Center for Plant Gene Research, Institute of Genetics and Developmental Biology, Chinese Academy of Sciences, Beijing, China

**Keywords:** elevated O_3_, ethylene, *Bemisia tabaci*, leaf senescence, amino acid, hormone-dependent defense

In the original article, the reference for Ludwików et al., 2014 was incorrectly written as Agnieszka et al., 2014. The correct reference is in the Reference list. The authors apologize for this error and state that this does not change the scientific conclusions of the article in any way.

The original article has been updated.

## Conflict of interest statement

The authors declare that the research was conducted in the absence of any commercial or financial relationships that could be construed as a potential conflict of interest.
